# Pre-Stroke Disability, Stroke Severity and Systemic Inflammation Outweigh Malignancy as Determinants of 90-Day Outcome After Acute Ischemic Stroke: A Single-Center Retrospective Cohort Study

**DOI:** 10.3390/brainsci16101095

**Published:** 2026-10-10

**Authors:** Justyna Zielińska-Turek, Jan Gajewski, Małgorzata Dorobek

**Affiliations:** 1 Department of Neurology, National Medical Institute of the Ministry of the Interior and Administration, Wołoska 137, 02-507 Warsaw, Poland; malgorzata.dorobek@pimmswia.gov.pl; 2 Department of Human Biology, Józef Piłsudski University of Physical Education in Warsaw, Marymoncka 34, 00-968 Warsaw, Poland; jan.gajewski@awf.edu.pl

**Keywords:** acute ischemic stroke, malignancy, pre-stroke disability, modified Rankin Scale, reperfusion therapy, C-reactive protein

## Abstract

**Background/Objectives**: Patients with acute ischemic stroke (AIS) and coexisting malignancy have worse outcomes, but it is unclear how much of this reflects malignancy itself rather than pre-stroke function, stroke severity and systemic inflammation. **Methods**: We retrospectively studied consecutive adults with MRI-confirmed AIS admitted to a neurology department in Warsaw, Poland (2020–2024): 130 with documented malignancy and 100 without. Because every patient with a pre-stroke modified Rankin Scale (mRS) score above 2 had a poor outcome by definition, the primary analysis was restricted to patients with pre-stroke mRS 0–2 (*n* = 161; 80 versus 81). Logistic models were adjusted sequentially for age, sex, admission NIHSS, pre-stroke mRS and log-transformed C-reactive protein (CRP). **Results**: Groups were comparable in age and vascular comorbidities. Good 90-day outcome was less frequent with malignancy (40.0% versus 58.0%; *p* = 0.028), but the association was no longer significant after adjustment for age, sex and admission NIHSS (odds ratio [OR] 1.55, 95% CI 0.69–3.51) and was null after adding pre-stroke mRS and log-CRP (OR 0.67, 0.25–1.81). Admission NIHSS and log-CRP were the consistent independent predictors. Return to within one mRS point of baseline was achieved by 41.5% versus 59.0% (*p* = 0.011). Among patients with pre-stroke mRS ≤ 2, intravenous thrombolysis rates were similar (22.5% versus 22.2%) and hemorrhagic transformation did not differ (17.4% versus 18.9%). **Conclusions**: Malignancy added little prognostic information beyond baseline function, stroke severity and systemic inflammation, although a clinically meaningful residual effect cannot be excluded. Among eligible patients, reperfusion appeared equally safe.

## 1. Introduction

Acute ischemic stroke (AIS) in patients with coexisting malignant neoplasms is associated with higher in-hospital mortality and an increased likelihood of discharge to a care facility rather than to home [1]. The most common malignancies associated with ischemic stroke include lung, prostate, breast, pancreatic and colorectal cancers [2]. Various mechanisms underlie the increased stroke risk in this population, the most prominent being hypercoagulability, nonbacterial thrombotic endocarditis (NBTE) and tumor embolism [3,4,5,6]. Notably, patients with solid organ malignancies are at higher risk for early complications, recurrent thromboembolic events, and increased morbidity and mortality following stroke [7,8].

Several mechanisms have been proposed to explain the excess stroke risk in patients with cancer, chiefly hypercoagulability, nonbacterial thrombotic endocarditis (NBTE) and tumor embolism, and these are most pronounced during periods of high disease activity [3,4,5,6]. Whether they operate in any individual patient depends on tumor type, stage and treatment, none of which is uniform across the populations studied. Reperfusion therapy is used less often in this group, although intravenous thrombolysis (IVT) is generally considered safe in carefully selected oncology patients and endovascular thrombectomy (EVT) yields outcomes broadly comparable to those in patients without malignancy [2,3,9,10,11]. Patients with cancer also face a higher risk of recurrent ischemic stroke, poorer functional outcomes and discharge to institutional care [2,12,13]. Cancer-associated stroke is increasingly recognized as a distinct clinical entity characterized by systemic inflammation, endothelial dysfunction and cancer-related hypercoagulability. Inflammatory biomarkers such as C-reactive protein (CRP), procalcitonin and D-dimer may reflect the biological interplay between malignancy and thromboembolic mechanisms, potentially influencing stroke severity and prognosis [13]. Elevated CRP at admission is an established independent predictor of mortality after ischemic stroke in the general stroke population [14].

A critical but underexamined consideration is that patients with malignancy differ from other stroke patients not only biologically but also functionally at baseline. Pre-stroke functional status, most commonly quantified with the modified Rankin Scale (mRS), is among the strongest determinants of post-stroke outcome and is used almost universally as an eligibility criterion for reperfusion therapy [15,16,17]. Patients with cancer frequently have reduced baseline function as a consequence of tumor burden, cachexia or oncologic treatment. If pre-stroke disability is not accounted for, the apparent prognostic effect of malignancy may be substantially confounded. Most previous cancer–stroke cohorts have not adjusted for pre-stroke mRS.In this retrospective single-center cohort study, we compared 90-day functional outcome, in-hospital mortality, access to reperfusion therapy and safety of reperfusion between patients hospitalized for AIS with and without comorbid malignancy. Our principal aim was to determine whether any association between malignancy and adverse outcome persists after sequential adjustment for stroke severity, pre-stroke functional status and systemic inflammation. We hypothesized that a substantial part of the excess adverse outcome attributed to malignancy would be explained by pre-stroke disability and inflammatory burden rather than by the presence of cancer itself.

## 2. Materials and Methods

### 2.1. Study Design and Population

**Figure 1:**
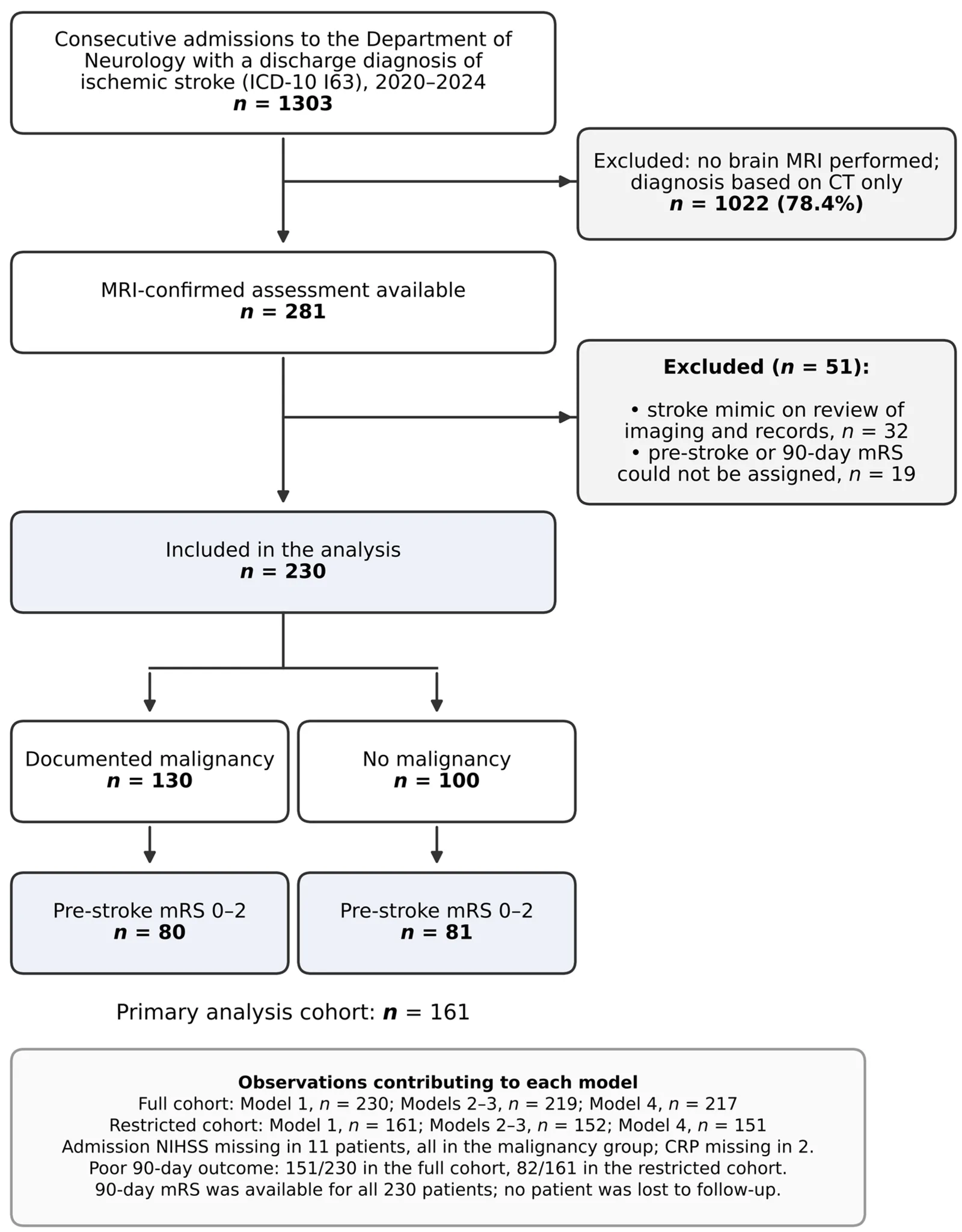
Flow of patients through the study. Of 1303 consecutive admissions to the Department of Neurology with a discharge diagnosis of ischemic stroke between January 2020 and December 2024, 281 underwent brain MRI; the remaining 1022 were diagnosed on CT alone and were not eligible for this study, which required diffusion-weighted confirmation. After exclusion of 32 stroke mimics and 19 patients in whom pre-stroke or 90-day mRS could not be assigned from the records, 230 patients were analyzed. Transient ischemic attack and intracranial hemorrhage are coded separately and did not enter the screened population. No patient contributed more than one admission. Arrows indicate the flow of patients; grey boxes denote excluded patients and blue-shaded boxes the analyzed cohorts. mRS, modified Rankin Scale; NIHSS, National Institutes of Health Stroke Scale.

This retrospective single-center cohort study was conducted at a neurology department in Warsaw, Poland, between January 2020 and December 2024. Consecutive adults admitted with a discharge diagnosis of ischemic stroke (ICD-10 I63) were identified from the hospital electronic medical record and radiological databases; 1303 admissions were screened. Because the study required imaging confirmation of the ischemic lesion, eligibility was restricted to patients who underwent brain MRI with diffusion-weighted imaging (DWI) and apparent diffusion coefficient (ADC) mapping (*n* = 281); the remaining 1022 patients were diagnosed on computed tomography alone. Patients in whom review of the imaging and clinical records did not confirm ischemic stroke were classified as stroke mimics and excluded (*n* = 32), as were patients whose records did not permit assignment of pre-stroke or 90-day mRS (*n* = 19). Transient ischemic attack and intracranial hemorrhage carry separate diagnostic codes and therefore did not enter the screened population. Each patient contributed a single index admission; no patient was admitted twice for ischemic stroke during the study period. The final cohort comprised 230 patients (Figure 1). The cohort was divided into two subgroups: patients with AIS alone (Group 1) and patients with AIS and coexisting malignancy (Group 2). All patients underwent brain magnetic resonance imaging (MRI) to confirm the diagnosis of AIS, defined by the presence of ischemic lesions on diffusion-weighted imaging (DWI) and apparent diffusion coefficient (ADC) maps. At admission, demographic data, vascular risk factors, malignancy type, laboratory findings and neurological assessments were recorded. A detailed medical history was obtained to document prior thrombotic events, including myocardial infarction, pulmonary embolism and deep vein thrombosis.

The comparison group comprised consecutive stroke admissions without any history of malignancy identified over the same period; no individual matching procedure was performed, and all eligible consecutive patients meeting inclusion criteria were included. Malignancy was defined as any histologically or radiologically confirmed malignant neoplasm documented in the medical history at the time of admission, irrespective of disease activity, stage or ongoing treatment, and we use the term documented malignancy throughout. The available records did not permit disease activity at the time of the stroke to be established reliably, and no attempt was made to distinguish active from remote disease; the implications of this are considered in the Discussion.

### 2.2. Variables and Outcomes

Stroke severity was assessed with the National Institutes of Health Stroke Scale (NIHSS) at admission and at discharge [18]. All standard contraindications to reperfusion therapy were assessed clinically in every patient, including recent major surgery, coagulopathy, thrombocytopenia, intracranial neoplasm or metastasis, therapeutic anticoagulation and uncontrolled hypertension. However, the only reasons for non-qualification recorded systematically in the study database were a pre-stroke mRS > 2 and arrival at the department outside the therapeutic time window. Analyses of treatment access were therefore performed both in the whole cohort and restricted to patients with pre-stroke mRS ≤ 2, the latter approximating eligibility on the recorded criterion alone rather than representing a complete eligibility assessment. Time from symptom onset to admission was not systematically recorded in the study database and could not be analyzed. Hemorrhagic transformation was assessed on follow-up imaging in patients who received IVT and/or EVT. The primary outcome was functional status at 90 days, measured with the mRS and dichotomized as good (0–2) versus poor (3–6). Secondary outcomes were death at 90 days (mRS 6), in-hospital death, receipt of reperfusion therapy, hemorrhagic transformation, discharge destination and length of hospital stay. Discharge destination was categorized as home, inpatient rehabilitation, long-term care facility, hospice or in-hospital death.

All patients underwent a structured clinical assessment on admission as part of routine departmental practice, performed by the admitting neurologist. This assessment includes the NIHSS, the Glasgow Coma Scale and the mRS reflecting the patient’s functional status in the week preceding stroke onset, together with chronic conditions, current medication, social and living circumstances, blood pressure, heart rate, body weight and temperature. Pre-stroke mRS was therefore recorded prospectively at the point of admission as part of clinical care, and was not assigned retrospectively for the purposes of this study. Because it was recorded on the day of admission, it necessarily preceded ascertainment of the 90-day outcome and cannot have been influenced by knowledge of it. Scoring was performed by the admitting neurologist on duty and was not blinded to the presence of a malignancy, which is documented in the admission history. Ninety-day mRS was ascertained from two sources, and the source used was recorded for every patient. The institution is a tertiary multi-specialty center comprising 28 clinical departments, an affiliated outpatient neurology clinic and an affiliated long-term care facility; patients therefore frequently re-presented within the same electronic medical record system, either for scheduled neurological follow-up, for care under another specialty, or as residents of the affiliated care facility. For these patients, functional status was extracted from the documentation generated closest to day 90, and this was the source for 208 of 230 patients (90.4%): 125 of 130 (96.2%) in the malignancy group and 83 of 100 (83.0%) in the comparison group. For the remaining 22 patients (9.6%), almost all of whom had been discharged home or to an external rehabilitation unit and had not re-presented, functional status at 90 days was obtained by telephone contact with the patient or a family member. Where no assessment fell precisely at day 90, the closest available record within ±30 days was used. Assessors were not blinded to malignancy status.

### 2.3. Statistical Analysis

Distributions were assessed with the Kolmogorov–Smirnov test and were significantly right-skewed. Continuous variables are therefore presented as medians with interquartile ranges (IQR) and compared using the Mann–Whitney U test; categorical variables are presented as counts with percentages and compared using Fisher’s exact test. CRP was log-transformed for regression analyses because of its skewed distribution.

Multivariable logistic regression was used to estimate the association between malignancy and outcome, with covariates entered in prespecified sequential blocks: (1) unadjusted; (2) age, sex and admission NIHSS; (3) additionally pre-stroke mRS; (4) additionally log-CRP. Because the dichotomized 90-day outcome is determined at baseline for patients with pre-stroke mRS > 2, none of whom can be classified as having a good outcome, the primary analysis was restricted to patients with pre-stroke mRS 0–2; the corresponding models in the whole cohort are reported as a secondary analysis to permit comparison with the published literature. As a second outcome anchored to each patient’s own baseline, we analyzed return to the pre-stroke functional level, defined as a 90-day mRS not exceeding the pre-stroke mRS (Δ mRS ≤ 0) and, as the primary definition for modelling, return to within one point (Δ mRS ≤ 1). Results are reported as odds ratios (OR) with 95% confidence intervals (CI). Analyses of reperfusion access were repeated in the subgroup meeting the institutional eligibility threshold (pre-stroke mRS ≤ 2). No imputation was performed; the number of observations contributing to each model is reported. A two-sided *p* < 0.05 was considered statistically significant. All analyses were conducted with SPSS version 28.0 (IBM Corp., Armonk, NY, USA) and verified independently.

## 3. Results

### 3.1. Baseline Characteristics

The study included 230 patients: 100 without malignancy (Group 1) and 130 with concomitant malignancy (Group 2). The two groups were comparable in age (median 76 vs. 74 years; *p* = 0.14), sex distribution, and prevalence of hypertension, diabetes mellitus, atrial fibrillation, chronic obstructive pulmonary disease, chronic kidney disease and current smoking (Table 1). Admission NIHSS did not differ significantly between groups (median 7 vs. 5; *p* = 0.16).

Two features clearly distinguished the groups. First, pre-stroke functional status was substantially worse among patients with malignancy (median mRS 2 vs. 1; *p* < 0.001), with 50 patients (38.5%) versus 19 (19.0%) having a pre-stroke mRS greater than 2 (*p* = 0.001). Second, inflammatory and coagulation markers were markedly elevated in the malignancy group: CRP 19.4 versus 3.2 mg/L, procalcitonin and D-dimer both *p* < 0.01, while leukocyte counts were comparable (Table 1).

Among the malignancies represented, breast cancer accounted for 18.46% of cases, followed by lung (16.15%), hematologic malignancies (14.61%), prostate (13.07%) and pancreatic cancer (8.46%). Less common types included colon and bladder cancer (each 7.69%), skin (3.84%), uterine (3.07%), gastric and laryngeal (each 2.31%) and other types (1.33%). No significant association was found between malignancy subtype and 90-day outcome or in-hospital mortality (*p* > 0.05).

### 3.2. Access to Reperfusion Therapy

**Figure 2:**
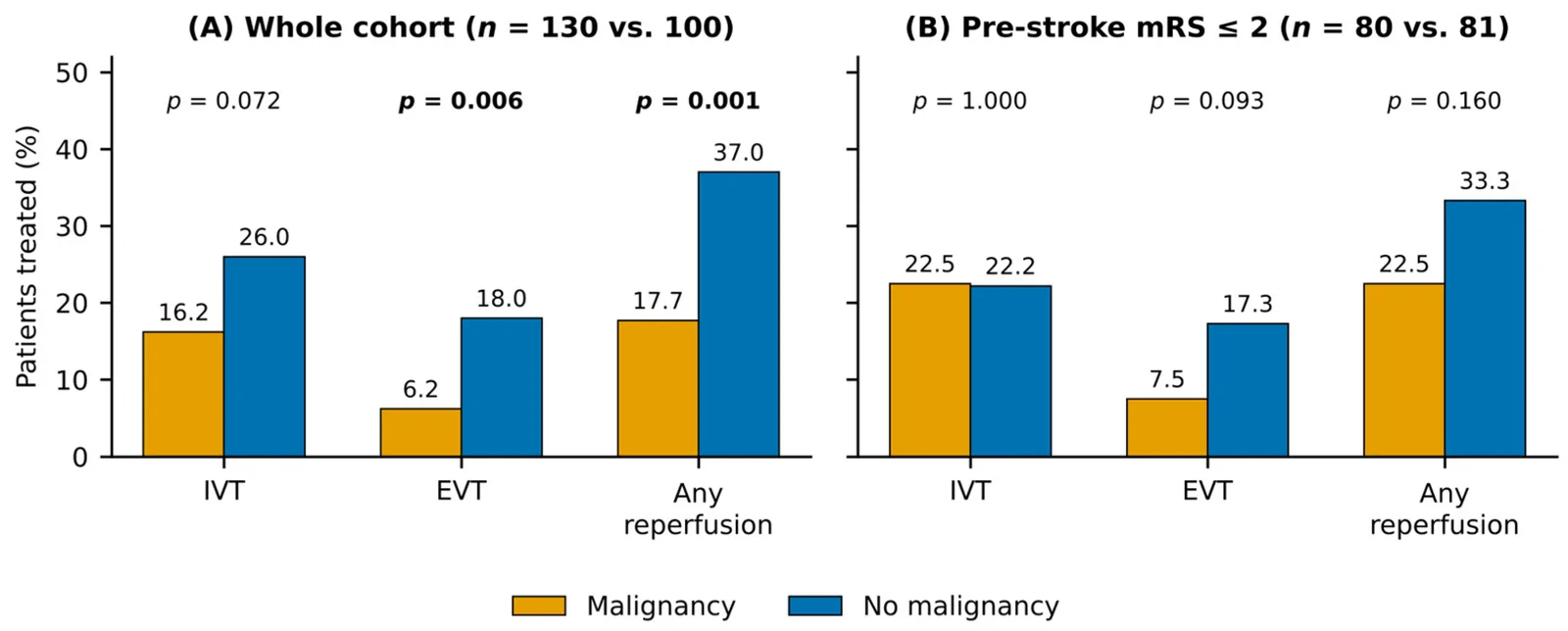
Rates of reperfusion therapy in patients with and without malignancy. (**A**) Whole cohort; (**B**) patients meeting the institutional eligibility threshold of pre-stroke mRS ≤ 2. Bars show the percentage of patients treated in each group; *p* values are from Fisher’s exact test; values in bold denote *p* < 0.05. EVT, endovascular thrombectomy; IVT, intravenous thrombolysis; mRS, modified Rankin Scale.

Across the whole cohort, patients with malignancy received reperfusion therapy less often than controls (23/130, 17.7% vs. 37/100, 37.0%; *p* = 0.001; Figure 2A). This difference was driven mainly by EVT (6.2% vs. 18.0%; *p* = 0.006) rather than IVT (16.2% vs. 26.0%; *p* = 0.07). In the malignancy group, 15 patients underwent IVT alone, 2 underwent EVT alone and 6 received combined IVT and EVT; in the non-malignancy group, 19 underwent IVT alone, 11 EVT alone and 7 combined therapy. When the analysis was restricted to patients meeting the institutional eligibility threshold of pre-stroke mRS ≤ 2, the difference attenuated and was no longer statistically significant (18/80, 22.5% vs. 27/81, 33.3%; *p* = 0.16; Figure 2B). IVT rates in this subgroup were similar (22.5% vs. 22.2%; *p* = 1.00), whereas EVT remained numerically lower in the malignancy group (7.5% vs. 17.3%; *p* = 0.09). In multivariable analysis adjusted for age, sex, admission NIHSS and pre-stroke mRS, malignancy remained associated with lower odds of receiving reperfusion therapy in the whole cohort (OR 0.42, 95% CI 0.21–0.83; *p* = 0.013); within the eligible subgroup the association was of similar magnitude but did not reach significance (OR 0.56, 95% CI 0.24–1.32; *p* = 0.18).

### 3.3. Safety of Reperfusion Therapy

Among the 60 patients who received IVT and/or EVT, hemorrhagic transformation occurred in 4 of 23 patients with malignancy (17.4%) and in 7 of 37 controls (18.9%; *p* = 1.00). In the malignancy group, hemorrhagic transformation followed combined IVT and EVT in 2 patients, IVT alone in 1 and EVT alone in 1; one of these patients died during hospitalization. Among patients who underwent reperfusion therapy, median NIHSS at admission was 8 in both groups.

### 3.4. Functional Outcome, Mortality and Discharge Destination

**Figure 3:**
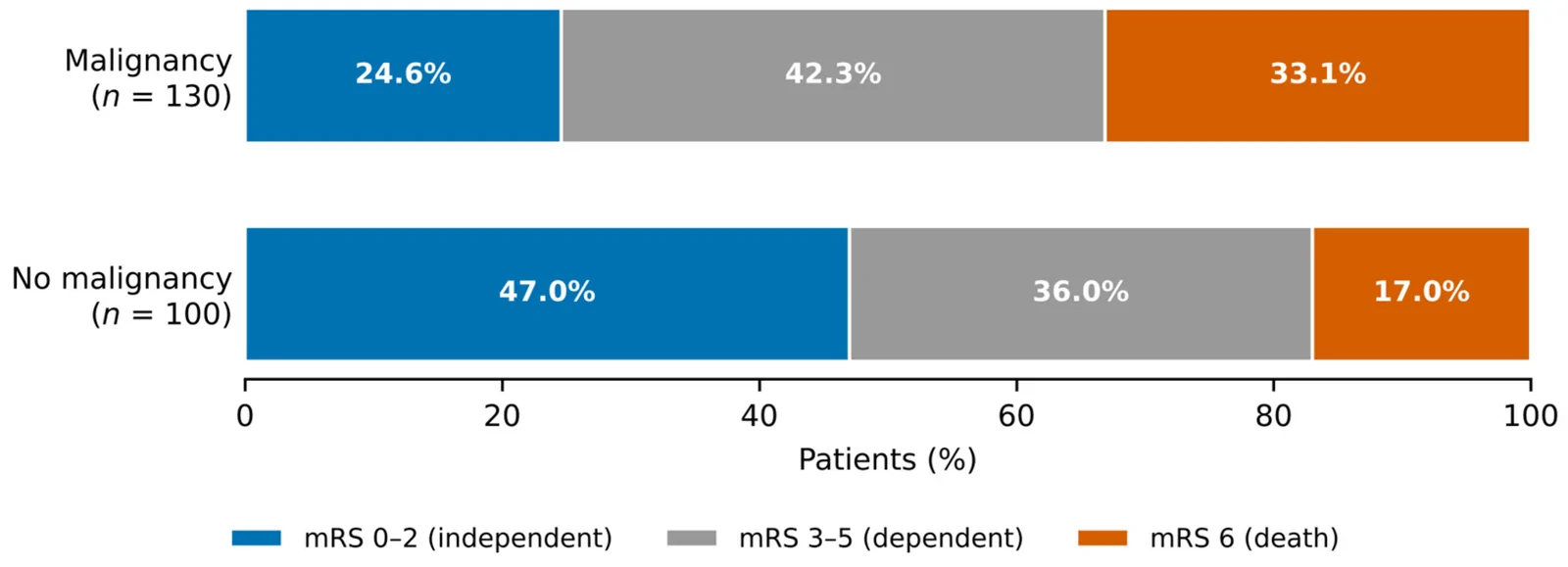
Distribution of functional outcome at 90 days according to malignancy status. Stacked bars show the proportion of patients who were functionally independent (mRS 0–2), dependent (mRS 3–5) or dead (mRS 6) at 90 days. mRS, modified Rankin Scale.

Crude outcomes were worse in the malignancy group across all measures (Table 2, Figure 3). Good 90-day outcome (mRS 0–2) was achieved by 32 patients (24.6%) with malignancy versus 47 (47.0%) without (*p* < 0.001), and 90-day death occurred in 43 (33.1%) versus 17 (17.0%; *p* = 0.006). In-hospital death occurred in 36 patients (27.7%) versus 16 (16.0%; *p* = 0.039). Patients with malignancy were more often discharged to a long-term care facility or hospice (21, 16.2% vs. 3, 3.0%; *p* < 0.001) and were referred for inpatient rehabilitation four times less frequently (4.6% vs. 20.0%; *p* < 0.001). Length of hospital stay did not differ (median 11 vs. 10 days; *p* = 0.18). Among patients who survived to discharge, NIHSS at discharge was similar in both groups (median 3 vs. 2; *p* = 0.11).

**Figure 4:**
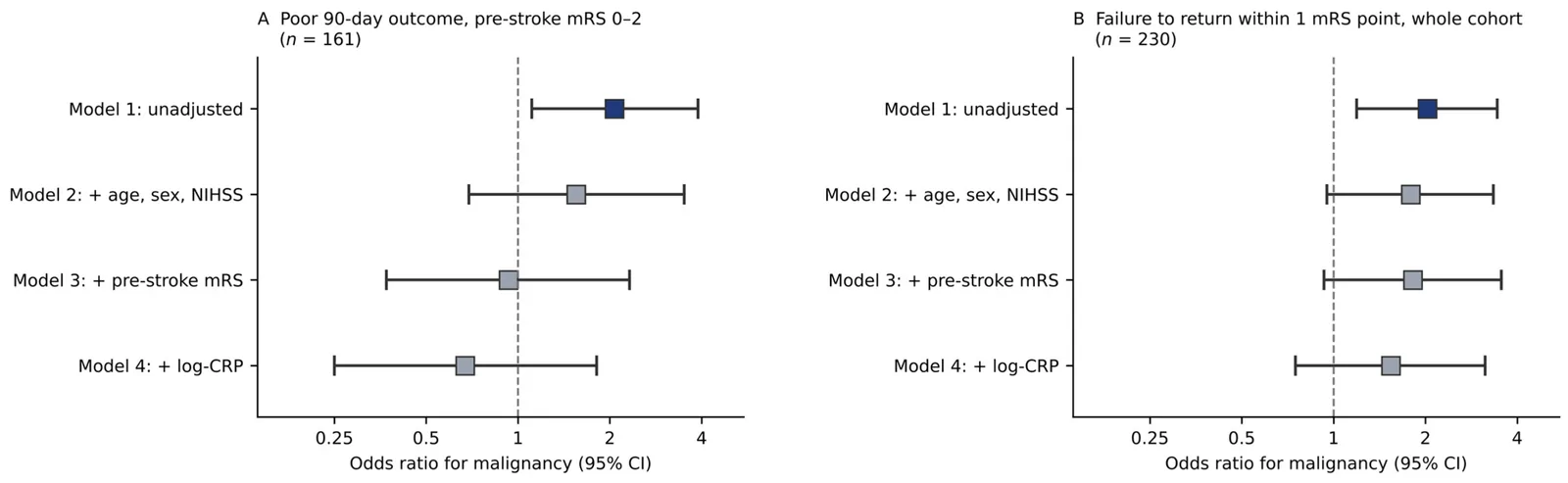
Sequential attenuation of the association between malignancy and outcome. (**A**) Poor 90-dayoutcome (mRS 3–6) in patients with pre-stroke mRS 0–2 (primary analysis; *n* = 161); (**B**) failure to return to within one point of the pre-stroke mRS in the whole cohort (*n* = 230). Squares denote odds ratios for malignancy and horizontal lines the corresponding 95% confidence intervals; dark blue squares indicate statistically significant estimates and grey squares non-significant estimates; the dashed line marks the null value and the x-axis is logarithmic. In both analyses, the crude association (Model 1) is no longer statistically significant after adjustment for age, sex and admission NIHSS (Model 2) and does not regain significance after further adjustment for pre-stroke mRS (Model 3) and log-CRP (Model 4). CRP, C-reactive protein; mRS, modified Rankin Scale; NIHSS, National Institutes of Health Stroke Scale.

**Figure 5:**
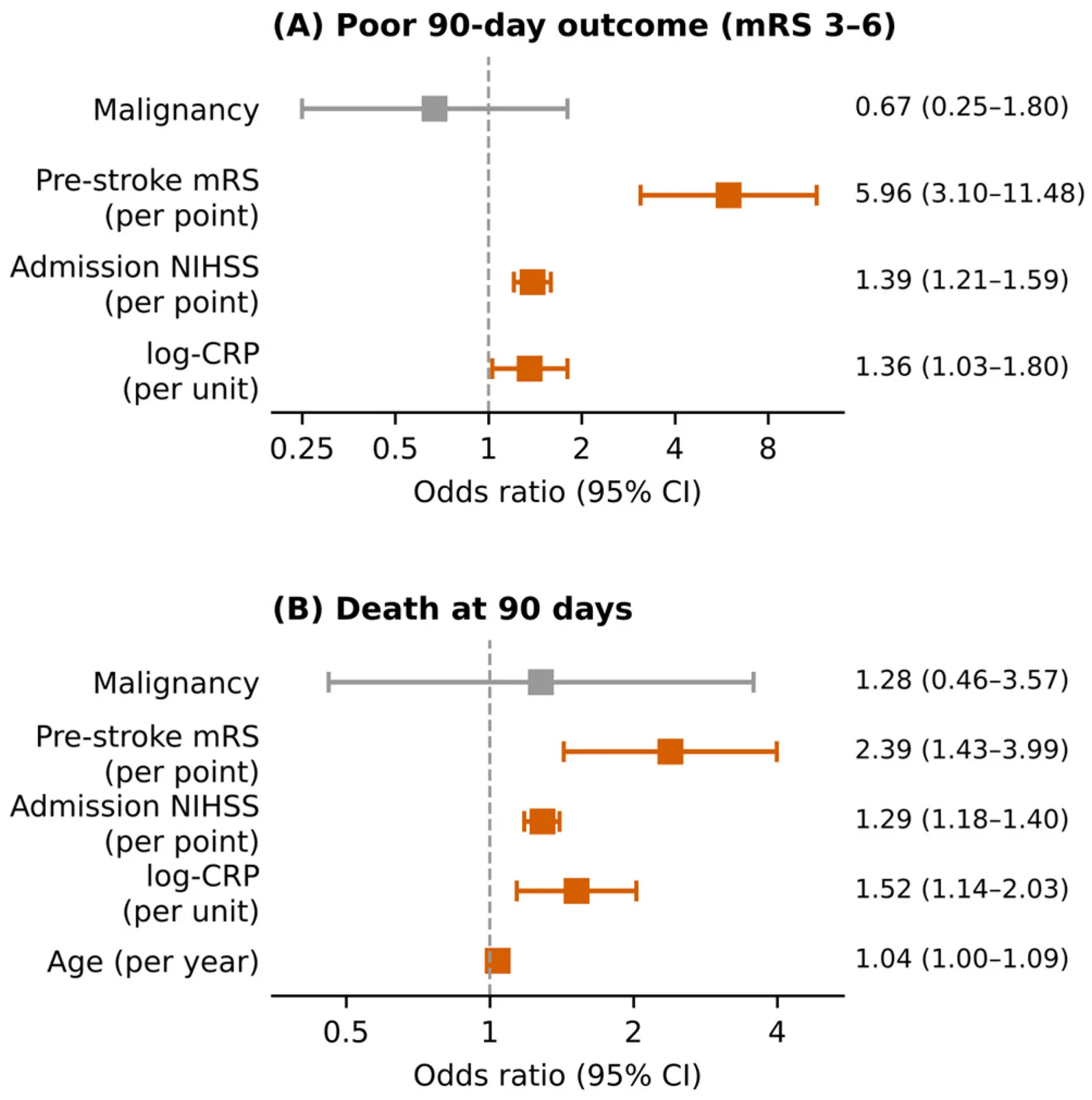
Independent predictors of outcome in the fully adjusted models. (**A**) Poor 90-day outcome (mRS 3–6); (**B**) death at 90 days. Squares denote odds ratios and horizontal lines 95% confidence intervals; grey indicates the non-significant estimate for malignancy and orange the statistically significant independent predictors; the dashed line marks the null value and the x-axis is logarithmic. CRP, C-reactive protein; mRS, modified Rankin Scale; NIHSS, National Institutes of Health Stroke Scale.

In sequential multivariable models for poor 90-day outcome in the whole cohort (Table 3; the corresponding models in the primary restricted cohort are shown in Figure 4A), the crude association with malignancy (OR 2.72, 95% CI 1.55–4.75) persisted after adjustment for age, sex and admission NIHSS (OR 2.54, 95% CI 1.22–5.29; *p* = 0.013), was attenuated to the null by the addition of pre-stroke mRS (OR 0.93, 95% CI 0.37–2.34; *p* = 0.88) and remained null after further adjustment for log-CRP (OR 0.67, 95% CI 0.25–1.80; *p* = 0.42). These intervals are wide and do not exclude odds of poor outcome more than twice as high in patients with malignancy. In the fully adjusted model, the independent predictors of poor outcome (Figure 5A) were pre-stroke mRS (OR 5.96, 95% CI 3.10–11.48; *p* < 0.001), admission NIHSS (OR 1.39, 95% CI 1.21–1.59; *p* < 0.001) and log-CRP (OR 1.36, 95% CI 1.03–1.80; *p* = 0.031). As set out in Section 3.5, the estimate for pre-stroke mRS in this model is inflated by the determinacy of the outcome in patients with pre-stroke disability, and these whole-cohort models should be read as secondary to the restricted analysis. The pattern for 90-day mortality was similar (Figure 5B). Malignancy was not independently associated with death in the fully adjusted model (OR 1.28, 95% CI 0.46–3.57; *p* = 0.63), whereas admission NIHSS (OR 1.29, 95% CI 1.18–1.40; *p* < 0.001), pre-stroke mRS (OR 2.39, 95% CI 1.43–3.99; *p* < 0.001), log-CRP (OR 1.52, 95% CI 1.14–2.03; *p* = 0.004) and age (OR 1.04, 95% CI 1.00–1.09; *p* = 0.037) all were.

### 3.5. Primary Analysis Restricted to Patients Without Pre-Stroke Disability

Of the 230 patients, 69 had a pre-stroke mRS above 2, and every one of them had a poor 90-day outcome (61/61 at mRS 3 and 8/8 at mRS 4); for these patients the primary outcome was determined at baseline. When pre-stroke mRS was entered categorically in the whole cohort, these strata were completely separated, and the coefficients for malignancy, age, sex, admission NIHSS and log-CRP converged to values identical to those obtained in the restricted cohort. The primary analysis was therefore restricted to the 161 patients with pre-stroke mRS 0–2, in whom the two groups were of near-equal size (80 with malignancy, 81 without).

Within this cohort, good 90-day outcome was achieved by 32 patients (40.0%) with malignancy versus 47 (58.0%) without (*p* = 0.028), and median 90-day mRS was 3 (IQR 2–4) versus 2 (1–3; *p* = 0.004). Death at 90 days occurred in 11 (13.8%) versus 7 (8.6%; *p* = 0.33). Age (74 vs. 71 years; *p* = 0.23) and admission NIHSS (6 vs. 4; *p* = 0.29) did not differ significantly, whereas CRP remained higher in the malignancy group (14.4 vs. 3.1 mg/L; *p* < 0.001). In sequential models (Table 4, Figure 4A), the crude association with malignancy (OR 2.07, 95% CI 1.11–3.89; *p* = 0.023) was no longer statistically significant after adjustment for age, sex and admission NIHSS (OR 1.55, 95% CI 0.69–3.51; *p* = 0.29), that is, before pre-stroke mRS entered the model, and was null thereafter (Model 3 OR 0.93, 95% CI 0.37–2.32; Model 4 OR 0.67, 95% CI 0.25–1.81; *p* = 0.43). The confidence interval in the fully adjusted model does not exclude odds of poor outcome up to approximately 1.8-fold higher in patients with malignancy. Independent predictors in Model 4 were admission NIHSS (OR 1.38, 95% CI 1.21–1.58; *p* < 0.001), pre-stroke mRS (OR 4.25, 95% CI 2.05–8.84; *p* < 0.001) and log-CRP (OR 1.34, 95% CI 1.01–1.78; *p* = 0.042). For 90-day mortality, malignancy was not associated with death either unadjusted (OR 1.69, 95% CI 0.62–4.59) or after full adjustment (OR 0.43, 95% CI 0.09–2.13), whereas admission NIHSS (OR 1.38, 95% CI 1.20–1.59) and log-CRP (OR 1.87, 95% CI 1.16–3.04; *p* = 0.011) remained independent predictors.

Return to the pre-stroke functional level was analyzed in the whole cohort. Return to baseline (Δ mRS ≤ 0) was equally frequent in the two groups (21/130, 16.2% vs. 15/100, 15.0%; *p* = 0.86), but this definition favours patients who were already disabled, in whom there is less room to deteriorate; in the corresponding model, pre-stroke mRS was protective (OR 0.35, 95% CI 0.20–0.61). Return to within one point (Δ mRS ≤ 1) was achieved by 54 patients (41.5%) with malignancy versus 59 (59.0%) without (*p* = 0.011). In sequential models for failure to return within one point (Figure 4B), the odds ratio for malignancy was 2.03 (95% CI 1.19–3.44; *p* = 0.009) unadjusted, 1.79 (0.95–3.34; *p* = 0.069) after age, sex and admission NIHSS, 1.82 (0.93–3.55) after pre-stroke mRS and 1.54 (0.75–3.14; *p* = 0.24) after log-CRP. On this baseline-anchored outcome, pre-stroke mRS was not an independent predictor (OR 0.89, 95% CI 0.62–1.28; *p* = 0.53), whereas admission NIHSS remained strongly predictive (OR 1.24, 95% CI 1.15–1.33; *p* < 0.001).

The estimate for malignancy was insensitive to how 90-day status was obtained. Restricting the fully adjusted model to the 208 patients ascertained from institutional documentation gave OR 0.69 (95% CI 0.25–1.90) in the whole cohort and OR 0.69 (0.25–1.89) in the restricted cohort, and entering ascertainment source as a covariate gave OR 0.72 (0.26–1.98). Pre-stroke mRS was available for all 230 included patients, so a sensitivity analysis restricted to patients with documented baseline function is identical to the primary analysis.

## 4. Discussion

Malignant neoplasms and acute ischemic stroke remain among the leading causes of mortality, particularly in older adults. With population aging and improving cancer survival, the coexistence of ischemic stroke and malignancy is increasingly encountered [19]. Approximately 10% of patients presenting with ischemic stroke have a concomitant malignancy, and cancer patients face an approximately 17% higher risk of ischemic stroke compared with the general population [19,20]. This elevated risk reflects multiple mechanisms, including cancer-associated hypercoagulability, coagulopathies, tumor-related embolization, and indirect complications such as systemic infection or vascular compression. Malignant tumors frequently induce a hypercoagulable state through release of procoagulant factors such as tissue factor and through platelet activation, resulting in excessive thrombin generation and platelet aggregation [21]. A characteristic pattern includes multifocal infarcts in different vascular territories accompanied by markedly elevated D-dimer levels [21]. The risk is greatest during periods of high cancer activity, when the prothrombotic state is most pronounced [1].

In our cohort, crude outcomes reproduced this well-established picture: patients with malignancy had a lower rate of good 90-day outcome, higher 90-day and in-hospital mortality, and a substantially greater need for institutional care after discharge. The principal finding of this study, however, is that this excess did not survive adjustment for baseline functional status. Pre-stroke mRS emerged as the single strongest determinant of 90-day outcome, with an approximately six-fold increase in the odds of dependency or death per point, and its inclusion in the model reduced the odds ratio for malignancy from 2.54 to 0.93. Systemic inflammation, indexed by CRP, contributed additionally and independently, consistent with meta-analytic evidence that elevated admission CRP predicts mortality after ischemic stroke irrespective of cancer status [14]. Malignancy itself carried no residual prognostic weight in the fully adjusted model.

This observation has a direct clinical implication. Prognostic pessimism toward stroke patients with cancer is common, and the crude outcome data appear to support it. Our findings suggest that what is largely being observed is the prognostic weight of baseline disability together with an inflammatory state, both of which are measurable at the bedside and neither of which is specific to malignancy. A patient with cancer who was functionally independent before the stroke and who does not have marked systemic inflammation had an outcome profile comparable to that of a patient without cancer. This may also explain why an apparently independent effect of malignancy has persisted in the literature: most previous cancer–stroke cohorts, including the large registry analyses [2,22,23,24,25,26], did not adjust for pre-stroke mRS.

Consistent with this interpretation, the safety of reperfusion did not differ between groups: hemorrhagic transformation occurred in 17.4% of treated patients with malignancy versus 18.9% of treated controls. Among patients meeting the institutional eligibility threshold of pre-stroke mRS ≤ 2, intravenous thrombolysis was delivered at an essentially identical rate in both groups, indicating that the overall treatment gap was driven predominantly by eligibility rather than by clinician reluctance. A residual difference in thrombectomy rates persisted after adjustment in the whole cohort and did not reach significance within the eligible subgroup. In our center, non-qualification for reperfusion was determined by only two criteria: pre-stroke mRS > 2 and arrival outside the therapeutic time window. Since pre-stroke mRS was accounted for in the models, the residual difference is most plausibly attributable to later presentation among patients with malignancy. This possibility is clinically important because delay is potentially modifiable: stroke symptoms in oncology patients may be misattributed to the underlying disease or to adverse effects of treatment, and patients residing in care facilities or with limited social support may be recognized and transported later. Because time from onset to admission was not recorded in our database, this explanation could not be tested and remains a hypothesis for prospective study. It is notable in this context that pre-existing disability has itself been questioned as a justified exclusion criterion, with registry data showing that a substantial proportion of previously dependent patients return to their pre-stroke functional level after thrombolysis or thrombectomy [16,17,27].

Higher in-hospital mortality and an increased likelihood of discharge to long-term care in patients with concomitant malignancy—particularly lung, pancreatic and colorectal cancers—have been reported previously [2,25,26,28,29,30]. The presence of metastatic disease further elevates the risk of in-hospital death, prolongs hospitalization, and reduces the probability of discharge home [25,26]. Our data are consistent with these observations at the descriptive level: patients with cancer were significantly more likely to require post-discharge institutional care [31] and were referred for inpatient rehabilitation four times less often. The latter finding deserves attention in its own right, as reduced access to rehabilitation may itself contribute to poorer functional trajectories independently of stroke biology. In contrast to prior reports, we observed no significant association between specific malignancy subtype and outcome, which may reflect the limited sample size and heterogeneity of tumor types represented [1,20,21,22,23,24].

The exposure in this study is documented malignancy, not active cancer, and this shapes what the estimate means. Our group includes patients under active oncologic treatment alongside patients in long-standing remission, and 14.6% had hematologic rather than solid tumors, in whom the thromboembolic mechanisms usually invoked in cancer-associated stroke are unlikely to be the operative ones. The null estimate we report is therefore an average across a heterogeneous population, and it is compatible with a real prognostic effect confined to patients with active disease that is diluted by those in remission. Cohorts that have separated the two support this reading: Merlino et al. found increased three-month mortality after intravenous thrombolysis in patients with active cancer but not in those with remote cancer, whose outcomes were indistinguishable from patients without cancer [32]. A cohort restricted to active disease could plausibly yield a different result from ours, and our findings should not be extended to that population.

Secondary prevention after ischemic stroke in patients with cancer remains critically important and must be tailored to the interplay between malignancy, its treatment and stroke mechanisms. The choice between antiplatelet therapy and anticoagulation is often challenging, as stroke in oncology patients is frequently cryptogenic and driven by cancer-related hypercoagulability [1]. When a conventional etiology is identified, such as carotid stenosis or atrial fibrillation, standard protocols apply, although coexisting prothrombotic mechanisms should be considered. In presumed cancer-related stroke—for example marantic endocarditis [33], disseminated intravascular coagulation or tumor embolism—anticoagulation is generally preferred, with low-molecular-weight heparin regarded as the agent of choice, reflecting its established superiority over vitamin K antagonists in cancer-associated venous thromboembolism [22,34].

The independent contribution of admission CRP to both poor functional outcome and death invites a mechanistic interpretation extending beyond concurrent infection or tumor burden alone. Systemic inflammation has been implicated in the regulation of perivascular fluid transport, and loss of aquaporin-4 polarization at astrocytic endfeet has been linked experimentally to impaired interstitial solute clearance and aggravated edema [35,36]. Glymphatic failure has been proposed as a final common pathway of neurodegenera-tion [37], cerebrospinal fluid influx contributes to acute ischemic tissue swelling [38], and post-ischemic inflammation amplifies secondary brain injury [39]. A patient presenting with a high inflammatory burden may therefore face not only a more severe lesion but also a less efficient clearance environment during recovery. This interpretation remains hypothesis-generating in our data, since CRP was measured once at admission and no imaging marker of glymphatic function was available; non-invasive indices such as the diffusion tensor image analysis along the perivascular space (DTI-ALPS) index [40] would allow the hypothesis to be tested prospectively in this population.

### Limitations

This study is retrospective and single-center, and its principal limitation is the way the cohort was assembled. Diffusion-weighted confirmation was required, and only 281 of 1303 patients admitted with ischemic stroke during the study period underwent MRI; the analyzed cohort therefore represents 17.7% of stroke admissions. The decision to perform MRI is a clinical one, and the factors that drive it—diagnostic uncertainty, suspected posterior circulation or cryptogenic stroke, younger age, and the patient’s ability to tolerate the examination—are not independent of prognosis. Our cohort is consequently not representative of unselected stroke admissions, and the absolute rates we report should not be read as population estimates. The comparison group was not individually matched, although consecutive enrollment and the observed balance of baseline characteristics mitigate this concern.

This selection may also operate differentially between the groups, and we consider this the more important concern. We had originally attributed the high proportion of patients with malignancy in our cohort (56.5%) to the tertiary referral profile of the institution. A more likely explanation is that patients with cancer are preferentially referred for MRI, since stroke in this population is more often cryptogenic or multi-territorial and since differentiation from metastatic disease is frequently required. If so, the two groups are not drawn from the source population in the same way, and residual confounding by the indication for MRI cannot be excluded. We are unable to test this with the available data, and it constitutes a limitation on the comparison itself rather than only on its generalizability. Against this, the requirement for MRI confers a corresponding strength: diagnostic certainty in the analyzed cohort is high, all 32 stroke mimics identified were excluded before analysis, and 90-day mRS was available for every one of the 230 patients, with no loss to follow-up. Pre-stroke mRS was recorded at admission as part of routine structured assessment, so its temporal precedence over the outcome is guaranteed by design rather than by recall, and knowledge of the 90-day result cannot have influenced it. Three limitations nevertheless remain. Scoring was performed in the course of clinical care by the admitting neurologist on duty rather than through a validated structured interview, and the mRS is known to have imperfect interrater agreement when applied in this way. Because each patient was assessed once by a single clinician, inter-rater agreement cannot be estimated retrospectively in this cohort. Finally, the assessment was not blinded to the presence of a malignancy. The 90-day mRS, by contrast, was ascertained retrospectively from the clinical record and is subject to misclassification depending on the granularity of the notes. The method of ascertaining 90-day status differed between groups: documentation held within the institution sufficed for 96.2% of patients with malignancy but only 83.0% of controls, the remainder requiring telephone contact. This reflects the greater likelihood that patients with cancer remained within the institutional system through the affiliated care facility, admission to another department, or death, but it means that the two groups were not characterized by identical means, and differential ascertainment cannot be formally excluded. Telephone-derived scores rest on patient or family report rather than on direct examination by the study team.

Comprehensive oncological data, including functional performance scales such as Karnofsky or ECOG status, were not consistently available, and disease activity at the time of stroke could not be established; the consequences of this for the interpretation of our estimate are discussed above. Detailed stroke mechanism classification using TOAST criteria and precise vessel occlusion location were unavailable for all patients. This matters more in this population than in an unselected stroke cohort, since cancer-associated stroke is disproportionately cryptogenic and frequently multi-territorial; without etiological classification we cannot determine whether the residual difference in thrombectomy rates reflects a different distribution of occlusion sites, nor whether the outcome patterns we observed track stroke mechanism. Admission NIHSS was missing for 11 patients, all of them in the malignancy group, which reduces the sample for the adjusted models asymmetrically. Occult malignancy may have been present in some patients classified as controls, potentially introducing misclassification bias. The number of patients receiving reperfusion therapy was small (23 and 37), so the comparison of hemorrhagic transformation had limited statistical power and cannot exclude a clinically meaningful difference of moderate size; this analysis should be regarded as reassuring rather than confirmatory. Although the institutional criteria for non-qualification were limited to pre-stroke mRS > 2 and arrival outside the therapeutic window, neither the criterion applied to each individual patient nor the time from symptom onset to admission was recorded in the study database; the residual difference in thrombectomy rates therefore could not be decomposed. Finally, CRP was measured once at admission and cannot separate concurrent infection, tumor burden and the stroke-related inflammatory response.

## 5. Conclusions

Among patients who were functionally independent or only mildly disabled before their stroke, those with documented malignancy had worse crude 90-day outcomes, but this difference was no longer detectable once age, sex and stroke severity were taken into account, and malignancy carried no independent prognostic weight after baseline function and systemic inflammation were added. Admission stroke severity and admission C-reactive protein were the consistent independent predictors of both poor functional outcome and death. The confidence intervals around our estimates do not exclude a clinically meaningful residual effect of malignancy, and this cohort was not powered to exclude one; what the data support is the narrower claim that malignancy adds little prognostic information once baseline function and stroke severity are known. Among patients meeting institutional eligibility criteria, intravenous thrombolysis was delivered at a similar rate and hemorrhagic transformation after reperfusion was no more frequent than in patients without cancer. These findings argue against prognostic pessimism based on the presence of a cancer diagnosis alone and support individualized assessment anchored on baseline function and inflammatory burden. Prospective multicenter studies incorporating structured pre-stroke functional assessment, stroke etiology classification and oncological staging—and, in particular, a validated definition of disease activity at the time of stroke—are needed to determine whether a residual effect exists in patients with active disease.

## Figures and Tables

**Table 1:** Baseline characteristics of the study population.

Characteristic	Malignancy (*n* = 130)	No Malignancy (*n* = 100)	*p*
Age, years	76 (69–82)	74 (63–82)	0.138
Male sex	63 (48.5)	44 (44.0)	0.509
Hypertension	101 (78.3)	82 (82.0)	0.511
Diabetes mellitus	34 (26.4)	30 (30.0)	0.556
Atrial fibrillation	33 (25.6)	29 (29.0)	0.653
COPD	8 (6.2)	3 (3.0)	0.356
Chronic kidney disease	17 (13.2)	13 (13.0)	1.000
Current smoking	17 (13.2)	18 (18.0)	0.357
Pre-stroke mRS, median	2 (1–3)	1 (1–2)	<0.001
Pre-stroke mRS > 2	50 (38.5)	19 (19.0)	0.001
Admission NIHSS	7 (3–13)	5 (3–10)	0.162
CRP, mg/L	19.4 (3.2–60.8)	3.2 (1.2–10.1)	<0.001
Procalcitonin, ng/mL	0.1 (0.1–0.3)	0.1 (0.0–0.1)	<0.001
D-dimer, ng/mL	1507 (874–4704)	1174 (472–2193)	0.006
Leukocytes, ×10^9^/L	8.3 (6.0–11.0)	8.5 (6.9–10.5)	0.545

Values are *n* (%) or median (IQR). COPD, chronic obstructive pulmonary disease; CRP, C-reactive protein; IQR, interquartile range; mRS, modified Rankin Scale; NIHSS, National Institutes of Health Stroke Scale.

**Table 2:** Treatment and clinical outcomes.

Variable	Malignancy (*n* = 130)	No Malignancy (*n* = 100)	*p*
IVT, all patients	21 (16.2)	26 (26.0)	0.072
EVT, all patients	8 (6.2)	18 (18.0)	0.006
Any reperfusion, all patients	23 (17.7)	37 (37.0)	0.001
IVT, pre-stroke mRS ≤ 2	18 (22.5)	18 (22.2)	1.000
EVT, pre-stroke mRS ≤ 2	6 (7.5)	14 (17.3)	0.093
Any reperfusion, pre-stroke mRS ≤ 2	18 (22.5)	27 (33.3)	0.160
Hemorrhagic transformation after reperfusion	4/23 (17.4)	7/37 (18.9)	1.000
Length of stay, days	11 (9–18)	10 (9–13)	0.175
Discharge home	64 (49.2)	61 (61.0)	0.084
Discharge to rehabilitation	6 (4.6)	20 (20.0)	<0.001
Discharge to LTC or hospice	21 (16.2)	3 (3.0)	<0.001
In-hospital death	36 (27.7)	16 (16.0)	0.039
90-day mRS, median	4 (3–6)	3 (2–4)	<0.001
Good outcome (90-day mRS 0–2)	32 (24.6)	47 (47.0)	<0.001
90-day death (mRS 6)	43 (33.1)	17 (17.0)	0.006

Values are *n* (%) or median (IQR). Hemorrhagic transformation is reported among patients who received reperfusion therapy. EVT, endovascular thrombectomy; IVT, intravenous thrombolysis; LTC, long-term care; mRS, modified Rankin Scale.

**Table 3:** Sequential logistic regression models for poor 90-day outcome (mRS 3–6).

Model	OR for Malignancy (95% CI)	*p*	*n*
Model 1: unadjusted	2.72 (1.55–4.75)	<0.001	230
Model 2: +age, sex, admission NIHSS	2.54 (1.22–5.29)	0.013	219
Model 3: +pre-stroke mRS	0.93 (0.37–2.34)	0.883	219
Model 4: +log-CRP	0.67 (0.25–1.80)	0.423	217

Independent predictors in Model 4: pre-stroke mRS OR 5.96 (3.10–11.48), *p* < 0.001; admission NIHSS OR 1.39 (1.21–1.59), *p* < 0.001; log-CRP OR 1.36 (1.03–1.80), *p* = 0.031. These models are reported as a secondary analysis; the primary analysis, restricted to patients with pre-stroke mRS 0–2, is given in Table 4. CI, confidence interval; CRP, C-reactive protein; mRS, modified Rankin Scale; NIHSS, National Institutes of Health Stroke Scale; OR, odds ratio.

**Table 4:** Sequential logistic regression models for poor 90-day outcome (mRS 3–6) in patients with pre-stroke mRS 0–2 (primary analysis).

Model	OR for Malignancy (95% CI)	*p*	*n*
Model 1: unadjusted	2.07 (1.11–3.89)	0.023	161
Model 2: +age, sex, admission NIHSS	1.55 (0.69–3.51)	0.290	152
Model 3: +pre-stroke mRS	0.93 (0.37–2.32)	0.877	152
Model 4: +log-CRP	0.67 (0.25–1.81)	0.428	151

Independent predictors in Model 4: admission NIHSS OR 1.38 (1.21–1.58), *p* < 0.001; pre-stroke mRS OR 4.25 (2.05–8.84), *p* < 0.001; log-CRP OR 1.34 (1.01–1.78), *p* = 0.042. This restricted cohort comprises the 161 patients (80 with malignancy, 81 without) in whom the dichotomized outcome is not determined at baseline; the corresponding models in the whole cohort are given in Table 3. CI, confidence interval; CRP, C-reactive protein; mRS, modified Rankin Scale; NIHSS, National Institutes of Health Stroke Scale; OR, odds ratio.

## Data Availability

The datasets generated and/or analyzed during the current study are not publicly available due to privacy and ethical restrictions but are available from the corresponding author on reasonable request.

## Abbreviations

The following abbreviations are used in this manuscript:

ADC	Apparent diffusion coefficient
AIS	Acute ischemic stroke
CI	Confidence interval
CRP	C-reactive protein
DWI	Diffusion-weighted imaging
EVT	Endovascular thrombectomy
IQR	Interquartile range
IVT	Intravenous thrombolysis
mRS	Modified Rankin Scale
MRI	Magnetic resonance imaging
NBTE	Nonbacterial thrombotic endocarditis
NIHSS	National Institutes of Health Stroke Scale
OR	Odds ratio
